# Risk factors for blood culture contamination in a tertiary hospital: a one-year retrospective analysis

**DOI:** 10.1186/s12879-026-12945-z

**Published:** 2026-02-21

**Authors:** Tuba İlgar, Aybegüm Özşahin

**Affiliations:** 1 Department of Infectious Diseases and Clinical Microbiology, Faculty of Medicine, Recep Tayyip Erdoğan University, Rize, Türkiye; 2 Department of Infectious Diseases and Clinical Microbiology, Recep Tayyip Erdoğan University Training and Research Hospital, Rize, Türkiye

**Keywords:** Blood culture contamination, Risk factors, Tertiary care hospital, Healthcare worker

## Abstract

**Background:**

Blood culture contamination is a significant problem that can lead to misdiagnosis, unnecessary antibiotic use, and increased workload. This study aimed to determine the blood culture contamination rates in a tertiary hospital and to evaluate the factors associated with contamination.

**Methods:**

This retrospective descriptive study included blood culture samples collected at a tertiary hospital between January 2023 and December 2023. Cultures from patients aged ≥ 18 years were evaluated. Growth of coagulase-negative staphylococci (CoNS) and other skin flora agents that were not considered clinically significant were classified as contamination. Contamination risk factors, including the sampling unit, sampling period, and healthcare personnel who collected the cultures, were evaluated.

**Results:**

A total of 7716 blood cultures from 2388 patients were included. 11.8% of the cultures were defined as contaminated. Contamination rates were found to be significantly higher in internal medicine wards (12.5%) and intensive care units (13.5%) compared to surgical wards (3.6%) (*p* < 0.001). In logistic regression analysis, the risk of contamination was found to be 3 times higher in blood cultures taken from internal medicine wards and 4.1 times higher in ICUs compared to surgical wards (*p* < 0.001 and *p* < 0.001, respectively). Blood cultures obtained by intern doctors (sixth-year medical students) were independently associated with contamination (OR = 1.68; *p* < 0.001). The risk of contamination was higher in blood cultures taken between July and December (OR = 1.23; *p* = 0.004).

**Conclusion:**

Blood culture contamination is a multifactorial problem influenced by factors such as the sampling unit and staff experience. These findings highlight the need for targeted quality improvement interventions, particularly structured training for newly rotating clinical staff.

**Clinical trial registration:**

Not applicable.

## Background

Blood culture is a critical diagnostic method used to identify causative agents in infectious diseases and to plan targeted therapy. However, improper sample collection can compromise the reliability of blood cultures and lead to the growth of microorganisms belonging to the skin flora, especially coagulase-negative staphylococci (CoNS). The growth of flora agents in blood cultures forces the clinician to distinguish whether the isolated bacteria are a true infectious agent or a false positive [1]. Identification of isolates, susceptibility testing, and repeated cultures may be required. This results in an increased workload and cost, as well as problems such as inappropriate antibiotic use, and prolonged hospital stays [2, 3].

It is recommended that blood culture contamination rates should be kept at a level of 1–3% [4]. However, it is reported that these rates exceed the recommended levels in many healthcare facilities [5]. Factors affecting contamination rates include variables such as the agent used in skin antisepsis, sterile technique, training, blood culture collection by phlebotomists, and the unit from which the sample is taken [6, 7].

In light of this information, the rate of contaminated blood cultures has a significant impact on patient management, antibiotic stewardship policies, and costs, and requires regular monitoring. This study aimed to determine blood culture contamination rates in a tertiary care hospital over a one-year period and to identify factors associated with contamination.

## Methods

This retrospective descriptive study included all blood culture samples taken at our tertiary hospital between January 2023 and December 2023. Data were obtained by scanning records from the hospital’s information management system.

### Study population

The study included patients aged 18 and over who had at least one blood culture taken during the specified dates. Culture results that had been entered into the laboratory information system by technicians but had not yet been formally validated and electronically approved by a microbiology specialist were excluded from the study.

### Working environment and blood culture collection practices

In our hospital, blood culture collection is conducted by nurses or intern doctors. Intern doctors are sixth-year medical students and begin actively working in clinics every year in July. While blood culture collection is conducted by nurses in some clinics, this responsibility belongs to intern doctors in other units. Intern doctors received training in blood culture collection as part of their medical school curriculum; no additional structured in-hospital blood culture collection training was routinely provided before the start of their clinical rotations. For nurses, blood culture collection and aseptic technique training was provided on an annual basis.

### Blood culture evaluation criteria

Blood culture bottles were incubated for five days in an automated continuous monitoring system (BACT/ALERT^®^ 3D, bioMérieux, France). Species-level identification of isolates obtained from bottles yielding a positive signal was performed using the VITEK^®^ 2 Compact system (BioMérieux, France).

For cultures yielding CoNS, patients’ medical records were reviewed in detail. CoNS isolates were classified as true pathogens only if they were considered clinically significant by the attending physician or infectious diseases consultant and if antimicrobial therapy was initiated accordingly. CoNS growths not regarded as clinically significant and not treated with antibiotics were classified as contamination. Cultures reported by the microbiology laboratory as skin flora microorganisms (e.g., diphtheroids, *Bacillus* spp., *Cutibacterium* spp.) without further species-level identification were classified as blood culture contamination. Growth of pathogenic organisms (e.g., Gram-negative bacteria, *Staphylococcus aureus*, *Enterococcus* spp., *Streptococcus* spp., and *Candida* spp.) was classified as true positive culture.

Blood culture sampling was performed according to routine clinical practice at our institution and not according to a study-mandated protocol. Repeated blood cultures were obtained at the discretion of the treating physician, particularly when contamination was suspected (e.g., CoNS growth) to help differentiate contamination from true infection, or in selected patients with true pathogens to assess clinical response.

### Data parameters

The following parameters were evaluated:

- Whether a repeated blood culture was obtained within seven days after a culture classified as contaminated (including skin flora microorganisms and CoNS growths not considered clinically significant and not treated)
- The units where blood culture samples were taken (Internal medicine wards, surgical wards, intensive care units (ICU))
- The professional title of the healthcare worker who took the blood culture in the units (Nurse, intern doctor)
- Period of blood culture collection (January – June or July – December)

### Statistical analysis

All data were evaluated using IBM SPSS V. 22.0 (Armonk, NY: IBM Corp). Categorical variables were expressed as numbers and percentages. The normality of the age variable was assessed using the Kolmogorov-Smirnov test; because age did not follow a normal distribution, it was presented as the median (minimum – maximum). Association between contamination and sampling unit, sampling period, and healthcare personnel who collected the cultures were evaluated using the Chi-square test or Fisher’s exact test, as appropriate Binary logistic regression analysis was performed to identify independent risk factors for blood culture contamination. As multiple blood cultures could be obtained from the same patient, a sensitivity analysis was performed by restricting the dataset to the first blood culture per patient and repeating the binary logistic regression analysis to assess the robustness of the main findings. A p value < 0.05 was considered statistically significant.

### Ethics approval and consent to participate

This study was conducted in accordance with the Declaration of Helsinki. The study was approved by the Recep Tayyip Erdoğan University non-interventional clinical research ethics committee, with decision number 2024/308 dated 19 December 2024. Due to the retrospective nature of the study and the use of anonymized, routinely collected data, the requirement for informed consent was waived by the Recep Tayyip Erdoğan University non-interventional clinical research ethics committee.

## Results

A total of 7944 blood culture results from 2472 patients were obtained during the defined study period. 44 patients were excluded from the study because they were under 18 years of age, and 163 cultures were excluded due to the lack of expert approval. In total, 7716 blood culture results from 2391 patients were included in the study. Of the patients, 980 (41%) were female. The median age was 68 years (range, 18–118 years). Of the 7716 blood cultures, 4835 (62.7%) were obtained from internal medicine wards, 2017 (26.1%) from intensive care units, and 864 (11.2%) from surgical wards. A total of 5719 (74.1%) cultures were collected by nurses and 1997 (25.9%) by intern doctors. Regarding the sampling period, 4143 (53.7%) cultures were obtained between January and June, and 3573 (46.3%) between July and December.

No growth was detected in 6230 (80.7%) of the blood cultures; therefore, positive growth was observed in 1486 (19.3%) cultures. The distribution of microorganisms isolated from blood cultures with positive growth is presented in Table 1. Among the 1486 positive cultures, 324 (21.8%) yielded skin flora microorganisms, and were classified as contaminants. Coagulase-negative staphylococci (CoNS) were isolated in 622 (41.9%) positive cultures. Among these, only 35 cultures from 10 patients were considered true infection agents based on physician assessment and initiation of antimicrobial therapy; the remaining CoNS isolates were classified as contaminants. Overall, 911 of 7716 blood cultures (11.8%) were classified as contaminated, corresponding to 61.3% of all positive cultures. Repeated blood cultures within seven days were performed in 273 (30%) of the contaminated cultures. Among these repeated cultures, 69 (25.3%) were again classified as contaminated, 186 (68.1) showed no growth, and 18 (6.6%) yielded clinically significant pathogens.

Contamination rates in blood cultures taken from patients followed in internal medicine wards and ICUs (12.5% and 13.5%, respectively) were statistically significantly higher than those collected from surgical wards (3.6%) (*p* < 0.001). Blood culture contamination rates were statistically significantly higher in those taken by intern doctors than nurses, and those taken between July and December compared to other months (< 0.001 and *p* = 0.002, respectively) (Table 2).

**Table 1:** Distribution of microorganisms isolated from blood cultures with positive growth

Blood culture result (*n* = 1486)	*n* (%)
Gram-negative bacteria	243 (16.4)
*Escherichia coli*	92 (6.2)
*Acinetobacter* spp.	43 (2.9)
*Klebsiella* spp.	33 (2.2)
*Pseudomonas* spp.	23 (1.5)
*Enterobacter* spp.	9 (0.6)
*Stenotophomonas maltophilia*	9 (0.6)
*Serratia marcescens*	7 (0.5)
*Proteus* spp.	5 (0.3)
*Haemophilus influenzae*	4 (0.3)
*Salmonella* spp.	4 (0.3)
Other*	14 (0.9)
Gram-positive bacteria	825 (55.5)
Coagulase-negative staphylococci	622 (41.9)
*Staphylococcus aureus*	105 (7.1)
*Enterococcus* spp.	74 (5.0)
*Streptococcus* spp.	20 (1.3)
*Granulicatella adiacens*	4 (0.3)
Fungi	94 (6.3)
Non-albicans candida	53 (3.6)
*Candida albicans*	41 (2.8)
Skin flora microorganisms	324 (21.8)

Percentages are calculated among positive cultures (*n* = 1486)

*Other: *Chryseobacterium indologenes*,* Brucella melitensis*,* Citrobacter* spp., *Sphingomonas paucimobilis*,* Burkholderia* spp., *Ralstonia insidiosa*,* and Morganella morganii*

**Table 2:** Distribution of contaminated and non-contaminated blood cultures according to sampling unit, healthcare worker, and period

		Non-contaminated cultures*	Contaminated cultures**	p-value
Sampling unit	Surgical wards	831 (96.2)_a_	639 (11.2)_a_	< 0.001
	Internal medicine wards	4229 (87.5)_b_	606 (12.5)_b_	
	Intensive care unit	1745 (86.5)_b_	272 (13.5)_b_	
Healthcare worker collecting the culture	Nurse	5120 (89.5)	599 (10.5)	< 0.001
	Intern doctor	1685 (84.4)	312 (15.6)	
Period of blood culture collection	January–June	3195 (89.4)	378 (10.6)	0.002
	July–December	3610 (87.1)	533 (12.9)	

Categories sharing the same superscript letter do not differ significantly in terms of contamination rates after Bonferroni correction (*p* > 0.05)

* Non-contaminated cultures include both culture-negative and true-positive cultures yielding clinically significant pathogens

** Contaminated cultures include skin flora microorganisms and coagulase-negative staphylococci growths not regarded as clinically significant and not treated

In the logistic regression analysis, when surgical wards were taken as a reference, the risk of contamination in blood cultures taken in internal medicine wards was found to be 3 times higher, and in ICUs it was 4.1 times higher (*p* < 0.001 and *p* < 0.001, respectively). Taking blood cultures by intern doctors and taking them between July and December were also found to be independent risk factors (Table 3).

In the sensitivity analysis restricted to the first blood culture per patient, internal medicine wards (OR = 2.53, 95% CI: 1.45–4.41) and intensive care units (OR = 3.16, 95% CI: 1.79–5.57) remained significantly associated with blood culture contamination. Cultures obtained by intern doctors were also independently associated with higher contamination risk (OR = 2.03, 95% CI: 1.49–2.77). Older age was a significant predictor (OR = 1.012 per year, 95% CI: 1.003–1.020), whereas sex and sampling period were not.

**Table 3:** Multivariable logistic regression analysis of factors associated with blood culture contamination

Variable		B	OR (95% CI)	*p* value
Sampling unit	Internal medicine wards*	1.10	3.00 (2.08–4.31)	< 0.001
	Intensive care unit*	1.41	4.11 (2.84–5.97)	< 0.001
Healthcare worker collecting the culture (Intern doctors**)		0.52	1.69 (1.43–2.00)	< 0.001
Period of culture collection (July–December^#^)		0.21	1.23 (1.07–1.42)	0.004

OR, odds ratio; CI, confidence interval. The model was statistically significant (Omnibus test χ²=121.5, *p* < 0.001). Hosmer–Lemeshow goodness-of-fit test *p* = 0.334, Nagelkerke R²=0.030

* Compared with surgical wards. ** Compared with nurses. ^#^ Compared with January–June

## Discussion

Blood culture contamination remains a persistent quality problem with significant implications for patient safety and antimicrobial stewardship. Inappropriate blood culture collection techniques may result in contamination with skin flora, forcing clinicians to distinguish true bacteremia from false-positive results. This diagnostic uncertainty often leads to unnecessary antibiotic exposure, prolonged hospitalization, additional diagnostic procedures, and increased healthcare costs [8]. However, in our institution, systemic antimicrobial therapy requires approval by an infectious diseases specialist. Therefore, unnecessary antibiotic exposure due solely to contaminated cultures is uncommon in our setting. Instead, the main downstream consequences of contamination in our hospital were the need for repeated blood cultures, additional clinical reassessment, increased laboratory workload, and higher healthcare costs. In the present study, the overall blood culture contamination rate was 11.8%, which is markedly higher than the 1–3% target recommended by international guidelines [4]. This finding highlights an important quality gap in blood culture practices at our institution. Notably, contamination rates increased during periods corresponding to the initiation of intern doctors’ clinical duties, and this association remained consistent in sensitivity analyses, supporting the robustness of our findings.

Contamination rates reported in the literature show significant differences between centers. While a study in a tertiary hospital in Nigeria reported that 10% of blood cultures were contaminated [9], and studies in the United States and Palestine reported 3-4.8% [10, 11], this rate was reported as only 1.9% in a teaching hospital in Saudi Arabia [12]. These differences are associated with factors such as blood culture collection protocols and the presence of specialized phlebotomy teams [13].

In our study, the risk of contamination was found to be higher in blood cultures taken in internal medicine wards and ICUs compared to surgical wards. Similarly, recent studies report higher contamination rates in ICUs due to factors such as higher workload, technical difficulties in blood culture collection, and difficulty in vascular access [14, 15]. Higher contamination rates are reported in critically ill patients, those with septic shock, comorbidities, and elderly patients [2, 16]. Tenderanda et al. [17] reported higher contamination rates in internal medicine units and ICUs. However, there are publications reporting higher contamination rates in surgical units [12]. In our study, higher contamination rates were also detected in internal medicine wards and ICUs. This may be due to the lower proportion of critically ill patients in surgical wards, the presence of more experienced staff, fewer blood culture collections, and more planned interventions.

It is noteworthy that the contamination rate in blood cultures taken by intern doctors was found to be significantly higher than that taken by nurses in our study. In the literature, it has been shown that the contamination rates in cultures taken by special blood collection teams, experienced phlebotomists, or laboratory personnel are significantly lower compared to nurses or ward personnel [18, 19]. Although our hospital does not have a special blood collection team, it is thought that the reason for the higher contamination rate in samples taken by intern doctors may be due to the fact that nurses are both more experienced in blood collection and receive more training on culture collection techniques. In addition, the fact that intern doctors start their active duty in clinics in July of each year has been associated with the higher contamination rate during the July-December period. In response to the elevated contamination rate identified in this study, a prospective quality improvement initiative has been planned. This initiative will include training on blood culture collection for intern doctors prior to the start of their clinical rotations, followed by monitoring contamination rates to assess the impact of this intervention.

The single-center and retrospective design of our study limits the generalizability of the results. However, the large sample size and the evaluation of independent risk factors through multivariate analysis are strengths of the study. Although multiple blood cultures from the same patient may introduce within-patient correlation, a sensitivity analysis restricted to the first culture per patient yielded results consistent with the main analysis, supporting the robustness of the findings.

## Conclusions

In conclusion, blood culture contamination represents a multifactorial quality issue in clinical practice, with sampling unit, personnel performing the procedure, and collecting period playing significant roles. Strengthening education programs, adhering to standardized blood culture collection protocols, and, where possible, establishing dedicated blood collection teams may contribute to reducing contamination rates and improving patient care.

## Data Availability

The datasets used and/or analyzed during the current study are not publicly available due to institutional data protection regulations and the inclusion of sensitive patient information, but are available from the corresponding author on reasonable request.

## Declarations

### Use of artificial intelligence tools

AI-based language tools (ChatGPT, OpenAI) were used solely to improve the fluency and intelligibility of the text.

## Abbreviations

CoNS: Coagulase-negative staphylococci
ICU: Intensive care unit
OR: Odds ratio
CI: Confidence interval
SPSS: Statistical Package for the Social Sciences
